# Tumoral LINE-1 hypomethylation is associated with poor survival of patients with intrahepatic cholangiocarcinoma

**DOI:** 10.1186/s12885-017-3595-8

**Published:** 2017-08-29

**Authors:** Seorin Jeong, Kyoungbun Lee, Xianyu Wen, Younghoon Kim, Nam-Yun Cho, Ja-June Jang, Gyeong Hoon Kang

**Affiliations:** 1 Laboratory of Epigenetics, Cancer Research Institute, Seoul National University College of Medicine, Seoul, South Korea; 2 Department of Pathology, Seoul National University College of Medicine, 28 Yongon-dong, Chongno-gu, Seoul, 110-744 South Korea; 3 Laboratory of Epigenetics, Cancer Research Institute, Department of Pathology, Seoul National University College of Medicine, Seoul, Korea

**Keywords:** Cholangiocarcinoma, Line-1, Methylation, Prognosis, Pyrosequencing

## Abstract

**Background:**

DNA methylation changes occurring in cancer cells are featured with both promoter CpG island hypermethylation and diffuse genomic hypomethylation. Long interspersed element-1 (LINE-1) is repeated in an interspersed manner with an estimated 500,000 copies per genome. LINE-1 has its CpG sites of the 5′ untranslated region methylated heavily in normal cells and undergoes demethylation in association with cancerization. However, little information is available regarding LINE-1 hypomethylation and its prognostic implication in intrahepatic cholangiocarcinomas.

**Methods:**

A total of 172 cases of intrahepatic cholangiocarcinomas were analyzed for their methylation levels at four CpG sites of LINE-1 using bisulfite pyrosequencing. We examined the relation between tumoral LINE-1 methylation level and clinicopathological features, including survival.

**Results:**

Tumor differentiation, lymphatic invasion, and T stage were associated with a low average methylation level of LINE-1 at the four CpG sites; LINE-1 methylation level tended to be lower in high-grade differentiation, lymphatic emboli, and higher T stage. LINE-1 hypomethylation was significantly linked with lower cancer-specific survival in patients with intrahepatic cholangiocarcinoma and was found to be an independent prognostic parameter.

**Conclusions:**

Our findings suggest that tumoral LINE-1 hypomethylation could be a molecular biomarker heralding poor prognosis of patients with intrahepatic cholangiocarcinoma. Our findings need to be validated in further study.

**Electronic supplementary material:**

The online version of this article (10.1186/s12885-017-3595-8) contains supplementary material, which is available to authorized users.

## Background

DNA methylation changes occurring in cancer cells are featured with regional promoter CpG island hypermethylation and generalized genomic hypomethylation. Promoter CpG island hypermethylation contributes to inactivation of tumor suppressor genes or tumor-related genes, whereas diffuse genomic hypomethylation is associated with chromosomal instability [1]. Repetitive DNA elements comprise approximately half of the human genome, and Long interspersed element-1 (LINE-1) retrotransposons comprise approximately 18% of the human genome [2]. The 5′ untranslated region sequence of LINE-1 has a high density of CpG dinucleotides, which are heavily methylated in normal cells but undergo hypomethylation in most tissue types of human cancer, including colorectal cancer [3, 4]. Since the study by Weisenberger et al. demonstrated a close correlation between genomic DNA methylation levels, determined by high-performance liquid chromatography, and LINE-1 DNA methylation levels determined by PCR-based measurement [5], LINE-1 methylation levels assessed by PCR-based methylation assays have been considered a surrogate marker for genomic methylation levels.

Intrahepatic cholangiocarcinoma (ICC) is the second most common primary liver cancer that arises from any portion of the intrahepatic biliary tree. ICC is a fatal disease because of its detection at a late stage in its course, frequent lymphovascular or perineural invasion, and lack of effective therapeutic modalities [6, 7]. Cancer staging and subsequent allocation to the optimal treatment approach is crucial for ICCs. However, none of the existing staging systems, including the 7th version of the American Joint Cancer Committee/Union for International Cancer Control (AJCC/UICC) staging system, fulfills the criteria for an optimal staging system [8]. The current version of the AJCC/UICC tumor, lymph node, metastasis (TNM) staging system for ICCs has been controversial for its predictive power of prognosis [9, 10] because a recent study by the Japanese Liver Cancer Study Group demonstrated no difference in overall survival between TNM stage II and III ICCs [10]. Although more work should be done to optimize the existing staging systems, molecular biomarkers associated with clinical outcome can help to predict tumor behavior and clinical outcome and need to be developed.

Studies have demonstrated that tumoral LINE-1 hypomethylation is associated with dismal clinical outcome of patients in many tissue types of human cancer, including colon cancer and gastric cancer [11–16]. Furthermore, an independent association of tumoral LINE-1 hypomethylation with poor prognosis of cancer patients has been demonstrated in the colon, stomach, esophagus, liver, lung, and brain [13, 16–19]. In the literature, however, no information is available regarding the prognostic implications of LINE-1 methylation status in ICCs. In the present study, we analyzed levels of LINE-1 methylation in ICC specimens using bisulfite pyrosequencing and examined whether LINE-1 methylation status was correlated with clinicopathological features including survival.

## Methods

### Patients

A total of 172 formalin-fixed archival tissue samples were obtained from patients who underwent surgical resection for ICC at the Seoul National University Hospital, Seoul, South Korea, from April 2005 to December 2012. Fifteen non-neoplastic gallbladder tissue samples were obtained from patients with chronic cholecystitis. Hilar cholangiocarcinomas, which arise from the left and right hepatic ducts at or near their junctions, were excluded from the study. Through meticulous histological examinations, combined hepatocellular carcinoma and cholangiocarcinoma were excluded from the study. The type of operative procedures included sectionectomy or segmentectomy in 46 patients (26.7%), lobectomy in 124 (72.1%), and total hepatectomy in 2 (1.2%). Among 172 patients, 6 (3.5%) received neoadjuvant chemotherapy and 47 (27.3%) received adjuvant chemotherapy and/or radiotherapy. Thirty-four (19.8%) patients received adjuvant chemotherapy and 13 (7.6%) patients received concurrent chemoradiation therapy after surgery. All cases were reviewed by experienced gastrointestinal pathologists (KBL and JJJ) to confirm the diagnosis of ICC and to re-evaluate histological findings and tumor-node-metastasis (TNM) stages according to the 4th edition 2010 WHO classification and the 7th edition 2009 AJCC/UICC staging system, respectively [20, 21]. Gross types of ICC were classified into three types according to gross appearance, including mass-forming (MF) type, periductal infiltrative (PI) type, and intraductal growth (IG) type [22, 23]. When more than one type was found in a tumor, the tumor was classified as mixed type. This retrospective study was approved by the Institutional Review Board at the Seoul National University Hospital (IRB No. H-1011-046-339).

### DNA extraction and bisulfite modification

Through microscopic examination, tumor areas in which 1) the tumor cells comprised >45% of total neoplastic and non-neoplastic cells and 2) represented the predominant histological type of the individual case were marked with a marker pen. For cases with ICC of mixed gross type, tumor areas with highest tumor density were marked in the individual cases. The corresponding areas were scraped from unstained tissue glass slides with a knife blade. Because epithelial cells are usually denuded in normal intrahepatic bile ducts of the formalin-fixed surgical specimens, cystic ducts of cholecystectomy specimens were taken as surrogates for normal controls. Cystic duct epithelia were scraped from the unstained tissue glass slides and collected into microtubes containing 50 μL of tissue lysis buffer and proteinase K. After incubation of the tubes for 48 h at 55 °C, the lysates were subjected to heating at 95 °C for 30 min. This prolonged heating was found to be necessary for lessening the formalin fixation-induced discrepancy in the measured value of LINE-1 methylation level [24]. With fixation of tissue samples in formalin solution, formaldehyde induces protein-DNA crosslinks and interstrand DNA crosslinks which may cause some difficulty in thermal and alkaline denaturation. Incomplete denaturation of double-stranded DNA results in potential under-conversion of non-methylated cytosines to uracils during bisulfite treatment, which might cause misleading results in the measured values of LINE-1 methylation for formalin-fixed tissue samples. In a previous study, we found that formalin fixation causes artificial increases in the measured value of LINE-1 methylation level, and that prolonged heat-treatment of DNA samples obtained from formalin-fixed tissue samples decreased the discrepancy in the measured values of LINE-1 methylation level between paired fresh-frozen and formalin-fixed tissue samples [24]. Following centrifugation of the tissue lysates, the supernatants were transferred into new tubes. DNA samples were subjected to bisulfite modification of DNA samples using the EZ DNA methylation kit (Zymo Research, Orange, CA, USA). LINE-1 methylation levels were measured using PCR pyrosequencing assay. The primers and PCR conditions were described previously [12]. The methylation level at each CpG site was the percentage of C nucleotides relative to the sum of C and T nucleotides at each CpG site. The four percentage values in the four serial CpG sites (nucleotide positions 328, 321, 318, and 306 of X58075 (GenBank)) were averaged and this mean value was taken as the overall LINE-1 methylation level in a given sample.

### Statistical analysis

Because LINE-1 methylation data followed a normal distribution, we used parametric tests to compare groups. However, when the following criteria were not met, we used both parametric tests and non-parametric tests: when two or more groups were compared, each group n should be greater than 15. Parametric tests (student t-test and ANOVA) were performed for comparison of two groups and three or more groups, respectively. Non-parametric tests (Mann-Whitney test and Kruskal-Wallis test) were further performed for the comparison of two groups and three or more groups, respectively, when one group was not greater than 15. The cancer-specific survival was calculated as the time from the date of surgery to the date of death by ICC. The data from patients who did not experience cancer-specific death were censored at the last follow-up visit to obtain the cancer-specific survival. The Kaplan-Meier log rank test and Cox proportional hazard method were used for survival analysis. For multivariate analysis, variables that were found to be significant in univariate analysis were included in the Cox proportional hazard model, and statistically significant variables were then selected by backward elimination. All *p* values were two-sided, and the statistical significance was set at *p* < 0.05. SPSS software (IBM SPSS Statistics version 23; Chicago, IL, USA) was used for all statistical analyses.

## Results

### Demographic and clinicopathological data

In total, 172 ICC patients underwent hepatic resection between 2005 and 2012. Of these patients, 147 patients (85.5%) presented with a single tumor. The male to female ratio was 121:51, and average age was 62.7 years (median, 63 years; range, 38–80 years). Gross type was MF type in 141 patients, PI type in 8 patients, IG type in 18 patients, and MF plus PI type in 5 patients. Stage grouping was stage I in 40, stage II in 37, stage III in 30, and stage IV in 65. Grading was well differentiated in 23, moderately differentiated in 94, and poorly differentiated in 55. Demographic and clinicopathological findings are summarized in Table 1.

**Table 1:** Relationship between LINE-1 methylation level and clinicopathological parameters

	No.	Average	SD	*P*-value
Sex				
M	121	75.48	7.173	0.233
F	51	76.97	8.216	
Age				
<64 years	87	75.97	8.274	0.936
≥64 years	85	75.87	6.674	
Gross type				
Mass forming	141	75.66	7.616	0.702 (ANOVA)
Periductal infiltrative	8	78.62	3.790	0.558 (KW)
Intraductal growth	18	76.73	7.110	
Mixed	5	76.16	10.837	
Multiplicity				
single	147	75.73	7.898	0.422
multiple	25	77.04	4.501	
T stage				
pT1	48	78.08	6.972	0.049 (ANOVA)
pT2a	38	73.08	9.417	0.012 (KW)
pT2b	14	76.12	4.010	
pT3	47	76.04	7.026	
pT4	25	75.74	6.622	
N stage				
pN0	133	75.96	7.861	0.899
pN1	39	75.78	6.223	
M stage				
pM0	161	76.17	7.593	0.095
pM1	11	72.26	4.972	0.035 (MW^a^)
TNM stage				
I	40	77.81	7.579	0.229
II	37	74.42	8.747	
III	30	76.25	7.801	
IV	65	75.45	6.406	
Differentiation				
Well	23	79.69	4.766	0.014
Moderate	94	75.95	6.857	
Poorly	55	74.28	8.906	
Neural invasion				
Absent	118	75.77	8.146	0.701
Present	54	76.25	5.921	
Lymphatic invasion				
Absent	102	76.92	7.200	0.034
Present	70	74.46	7.749	
Vascular invasion				
Absent	95	76.44	8.141	0.396
Present	77	75.27	6.631	0.047 (MW^a^)
Chronic liver disease				
Absent	130	75.95	7.497	0.936
Present	42	75.84	7.617	
Chronic biliary disease				
Absent	159	75.93	7.679	0.962
Present	13	75.82	5.108	

^a^Mann-Whitney

### Relationship between LINE-1 methylation level and clinicopathological features

**Fig. 1:**
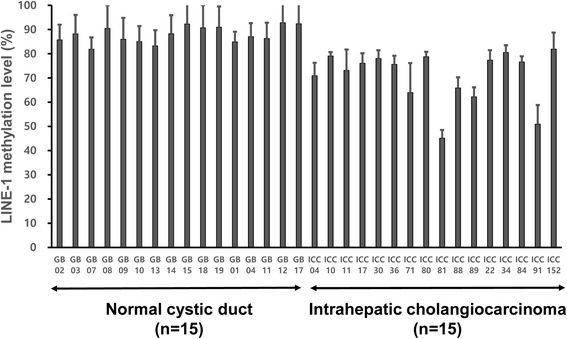
LINE-1 methylation level was significantly lower in tumor tissues of intrahepatic cholangiocarcinoma (*n* = 15) than in cystic duct epithelia of non-neoplastic gallbladder (*n* = 15) (student t-test, *p*-value <0.001)

The LINE-1 methylation level was significantly lower in ICC tissue samples than in normal gallbladder tissue samples (Fig. 1). Tumoral LINE-1 methylation levels were not different in ICCs between male and female patients and between younger and older patients (<64 years old and ≥64 years). No association was found between tumoral LINE-1 methylation levels and gross types. Tumoral LINE-1 methylation levels were not different between ICCs of single-tumor and multiple-tumor types. However, a significant difference was noted between well-differentiated ICCs and moderately or poorly-differentiated ICCs: well-differentiated ICCs showed higher methylation levels than those of moderately or poorly differentiated ICCs. Tumoral LINE-1 methylation levels tended to be higher in T1 stage than in T2b or higher T stages. No difference in LINE-1 methylation levels was found between ICCs of N0 and N1, or between ICCs of M0 and M1. While LINE-1 methylation levels were significantly lower in ICCs with lymphatic tumor emboli than in ICCs without lymphatic tumor emboli, no significant difference was noted between ICCs with and without venous tumor emboli and between ICCs with and without perineural invasion.

### Relationship between tumoral LINE-1 methylation status and cancer-specific survival of patients with ICC

**Fig. 2:**
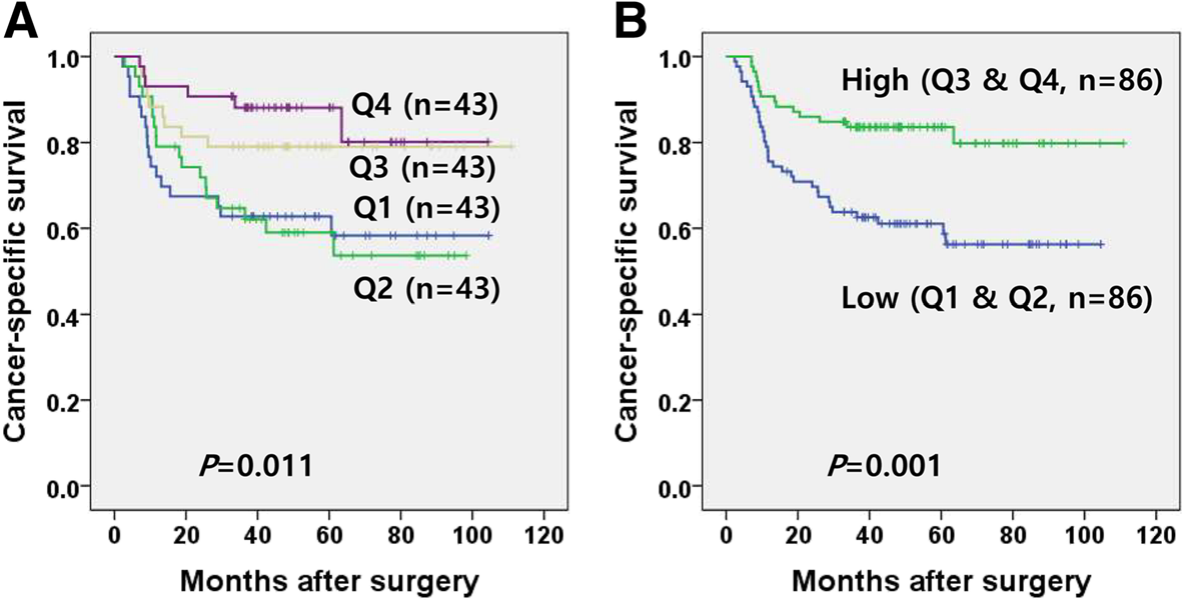
Kaplan-Meier survival analysis with log rank test. ICC patients were grouped into 4 quadrants according to their tumoral LINE-1 methylation levels. Q1, 2, 3, and 4 are ordered by increasing methylation level of LINE-1. Cancer-specific survival was lower in patients with Q1 or Q2 ICC than in patients with Q3 or Q4 ICC (**a**). When ICC patients were grouped into low LINE-1 methylation status (Q1 and 2) and high LINE-1 methylation status (Q3 and 4,), Cancer-specific survival was significantly lower in the low methylation status group than in the high methylation status group (**b**)

When ICCs were grouped into four quadrants (Q1, Q2, Q3, and Q4 in order of increasing level of LINE-1 methylation) based on their tumoral LINE-1 methylation levels, Q1 and Q2 exhibited significantly lower cancer-specific survival than that of Q3 and Q4 (Fig. 2). As displayed in the Kaplan-Meier survival curve, cancer-specific survival curves of Q1 and Q2 are similar, while cancer-specific survival curves of Q3 and Q4 are similar. Thus, ICCs were further grouped into low methylation status subgroup (Q1 and Q2) and high methylation subgroup (Q3 and Q4). Low LINE-1 methylation status was associated with worse cancer-specific survival in Kaplan-Meier survival analysis. In addition to low LINE-1 methylation status, T staging, N staging, lymphatic emboli, perineural invasion, and histological differentiation were included into a multivariate analysis which revealed that a low LINE-1 methylation status was independently associated with worse cancer-specific survival in patients with ICC (Tables 2 and 3).

**Table 2:** Univariate survival analysis of LINE-1 methylation level and clinicopathological parameters with respect to overall survival

Parameters		Hazard ratio (95% C.I.)	*P*-value
LINE-1 methylation status			0.017
	Q4 (*n* = 43)	Reference	
	Q3 (*n* = 43)	1.575 (0.561–4.602)	0.320
	Q2 (*n* = 43)	3.369 (1.328–8.551)	0.011
	Q1 (*n* = 43)	3.400 (1.349–8.570)	0.009
LINE-1 methylation status	Q3, Q4 (*n* = 86)		
	Q1, Q2 (*n* = 86)	2.643 (1.443–4.482)	0.002
pTNM staging			0.145
	I (*n* = 43)	Reference	
	II (*n* = 38)	1.636 (0.739–3.622)	0.224
	III (*n* = 30)	0.543 (0.173–1.709)	0.297
	IV (*n* = 61)	1.602 (0.768–3.342)	0.209
T staging			0.044
	pT1 (*n* = 48)	Reference	
	pT2a (*n* = 38)	1.809 (0.866–3.777)	0.114
	pT2b (*n* = 14)	2.344 (0.929–5.913)	0.071
	pT3 (*n* = 47)	0.941 (0.420–2.107)	0.883
	pT4 (*n* = 25)	0.433 (0.123–1.524)	0.192
N staging	pN0 (*n* = 133)		
	pN1 (*n* = 39)	2.328 (1.304–4.157)	0.004
M staging	pM0 (*n* = 161)		
	pM1 (*n* = 11)	1.482 (0.533–4.118)	0.451
Gross type			0.076
	Mass forming (*n* = 141)	Reference	
	Periductal infiltrative (*n* = 8)	0	0.964
	Intraductal growth (*n* = 18)	0.134 (0.018–0.971)	0.047
	Mixed (*n* = 5)	2.672 (0.827–8.638)	0.101
Lymphatic emboli	Absent (*n* = 102)		
	Present (*n* = 70)	2.519 (1.429–4.438)	0.001
Vascular invasion	Absent (*n* = 95)		
	Present (*n* = 77)	1.111 (0.634–1.945)	0.714
Perineural invasion	Absent (*n* = 118)		
	Present (*n* = 54)	0.386 (0.181–0.822)	0.014
Tumor border	Expanding (*n* = 32)		
	Infiltrative (*n* = 140)	2.133 (0.846–5.375)	0.108
Tumor differentiation			0.083
	Well (*n* = 23)	Reference	
	Moderate (*n* = 94)	9.405 (1.283–68.943)	0.027
	Poor (*n* = 55)	9.671 (1.290–72.518)	0.027
Chemotherapy and/or radiotherapy	No (*n* = 120)		
	Yes (*n* = 52)	0.979 (0.534–1.792)	0.944

**Table 3:** Multivariate survival analysis of LINE-1 methylation level and clinicopathological parameters with respect to overall survival

		Hazard ratio (95% C.I.)	*P*-value	Hazard ratio (95% C.I.)	*P*-value
LINE-1 methylation level^a^	Q3, Q4 (*n* = 86)				
	Q1, Q2 (*n* = 86)	2.643 (1.443–4.482)	0.002	2.248 (1.205–4.196)	0.011
N staging	pN0 (*n* = 133)				
	pN1 (*n* = 39)	2.211 (1.176–4.157)	0.014	2.749 (1.491–5.066)	0.001
T staging			0.025		0.130
	pT1 (*n* = 48)	Reference		Reference	
	pT2 (*n* = 52)	1.943 (0.979–3.855)	0.058	0.555 (0.211–1.458)	0.232
	pT3 (*n* = 47)	0.941 (0.420–2.105)	0.882	0.363 (0.121–1.091)	0.071
	pT4 (*n* = 25)	0.433 (0.123–1.523)	0.192	0.216 (0.053–0.889)	0.034
Gross type			0.076		0.064
	Mass forming (*n* = 141)	Reference		Reference	
	Periductal infiltrative (*n* = 8)	0	0.964	0	0.972
	Intraductal growth (*n* = 18)	0.134 (0.018–0.971)	0.047	0.123 (0.017–0.910)	0.040
	Mixed (*n* = 5)	2.672 (0.827–8.638)	0.101	2.878 (0.854–9.694)	0.088
Lymphatic emboli	Absent (*n* = 102)				
	Present (*n* = 70)	2.519 (1.429–4.438)	0.001	2.720 (1.168–6.337)	0.020
Perineural invasion	Absent (*n* = 118)				
	Present (*n* = 54)	0.386 (0.181–0.822)	0.014	0.373 (0.162–0.860)	0.021
Differentiation			0.083		0.638
	Well (*n* = 23)	Reference			
	Moderate (*n* = 94)	9.405 (1.283–68.943)	0.027		
	Poor (*n* = 55)	9.671 (1.290–72.518)	0.027		

^a^Regardless of whether adjuvant and/or neoadjuvant therapy was included or not in the multivariate analysis, the hazard ratio of LINE-1 methylation level did not change

## Discussion

Relationships between low tumoral LINE-1 methylation status and poor survival have been demonstrated in several types of gastrointestinal tract malignancies, including esophageal squamous cell carcinoma, gastric adenocarcinoma, and colorectal adenocarcinoma. However, no study is available regarding the relationship between tumoral LINE-1 hypomethylation and survival of patients with ICC. In the present study, we have for the first time demonstrated a close association between low tumoral LINE-1 methylation status and poor survival of patients with ICC. In our previous study, levels of LINE-1 methylation were demonstrated to be lower in extrahepatic cholangiocarcinomas (ECCs) than in normal bile ducts, and the timing of tumoral LINE-1 hypomethylation is a late event during multistep carcinogenesis of ECC [25]. However, our previous study did not analyze the relationship between tumoral LINE-1 methylation status and survival of patients with ECC. In the present study, findings indicate that tumoral LINE-1 hypomethylation might be an independent parameter heralding poor prognosis in patients with ICC.

Many tissue types of human cancer have demonstrated close associations between tumoral LINE-1 hypomethylation and poor prognosis of patients with the specific tissue type of cancer. However, no satisfactory explanation has been provided regarding the reason why tumoral LINE-1 hypomethylation status contributes to the aggressive behavior of the tumor, which is the same case in ICCs. Several speculations might be made regarding the mechanism by which tumoral LINE-1 hypomethylation contributes to poor survival in patients with ICC. Decreased methylation level of LINE-1 might lead to increased genomic instability through enhanced non-homologous recombination and subsequent chromosomal instability, increased retrotransposon activity and subsequent random insertional mutation, or decreased mRNA expression of genes harboring anti-directional LINE-1 s in their intron sequences. Such an enhanced genomic instability might cause an increased expression of proto-oncogenes or a decreased expression of tumor-suppression genes, which might contribute to increased aggressiveness of ICCs. However, the exact mechanism by which tumoral LINE-1 hypomethylation contributes to the aggressive behavior is unknown.

TNM system is used to stage ICCs, and staging helps to guide treatment decisions. However, the prognostic power of the current version TNM staging system for ICCs is dubious because in a recent study, overall survival was not different between stage II and III ICCs [10]. Because of the weak discriminating power of the current TNM staging system, it is necessary to develop molecular markers that can predict tumor behavior and help to predict the risk of recurrence after the surgery, in order to plan more effective cancer treatments. In a recent next-generation sequencing-based study, a clustering analysis of global gene expression levels was shown to predict prognosis of patients with biliary tract cancer [26]. The clustering by gene expression signature was associated with the distribution of driver gene alterations. ICCs belonging to a cluster with a high frequency of mutations in *BAP1*, *IDH1*, or *NRAS* tended to exhibit better clinical outcomes compared with ICCs belonging to two clusters with a high frequency of mutations in *TP53*, *KRAS*, or *SMAD4*. However, the clustering analysis of global gene expression levels should be validated in an independent study for its usefulness for prediction of prognosis. In the literature, however, no single molecular markers, except for immunohistochemical markers, have been demonstrated to be closely associated with clinical outcome of patients with ICC. Our findings indicated that tumoral LINE-1 hypomethylation status was closely associated with poor prognosis in patients with ICC and that tumoral LINE-1 hypomethylation was an independent biomarker heralding poor prognosis in patients with ICC. However, this finding should be validated in an independent set of ICCs.

Because the present study did not perform laser capture microdissection, a concern may well be raised over whether variable amounts of immune and stromal cells contained in the dissected tumor areas may affect the analysis of LINE-1 methylation level in ICC tumor samples. After we estimated the ratio of non-neoplastic cells in tumor areas which were marked for manual dissection, we analyzed the relationship between LINE-1 methylation level and stroma ratio of tumor area and found no significant correlation between them (Pearson correlation coefficient, 0.098 (*p*-value = 0.199); Spearman correlation coefficient, 0.103 (*p*-value = 0.178)) (see Additional file 1). For comparison of means, four subsets of ICCs according to percentage of non-neoplastic cells (<10%, 10–19%, 20–30%, >30%) were compared regarding the distribution of LINE-1 methylation level and no significant difference was seen in LINE-1 methylation level between the subsets (*p*-value by Kruskal-Wallis method, 0.242; *p*-value by ANOVA test, 0.527). For survival analysis, we grouped ICC cases into two subsets according to stroma cell ratio (≤15% (*n* = 90), and, >15% (*n* = 82)) and then evaluated prognostic potential of low methylation status of LINE-1 in each subsets. Regardless of stroma ratio status, prognostic significance of low methylation status of LINE-1 was seen in patients with ICC (see Additional file 2).

In the present study, because of the association between tumoral LINE-1 methylation level and lymphatic emboli, both of which were independent prognostic parameters in ICCs, we expected to develop combinatory markers that are superior in prognostic power to each alone. For this aim, ICCs were divided into two groups (ICCs with lymphatic emboli vs. without lymphatic emboli) according to lymphatic emboli. Then, multivariate analysis was performed in patients with ICCs to elucidate whether a combination of both parameters, LINE-1 methylation and lymphatic embolus statuses, would contribute to identification of a subgroup of ICCs with poor prognosis. Compared with ICCs with high tumoral LINE-1 methylation status and no lymphatic tumor emboli, ICCs with low tumoral LINE-1 methylation status and lymphatic tumor emboli showed a hazard ratio of 3.609 (1.639–7.945), whereas ICCs with low tumoral LINE-1 methylation status and no lymphatic tumor emboli harbored a hazard ratio of 0.858 (0.303–2.430) (Table 4).

**Table 4:** Multivariate survival analysis of combinatory LINE-1 methylation level and lymphatic embolus status and clinicopathological parameters with respect to overall survival

Parameters		Hazard ratio (95% C.I.)	*P*-value
LINE-1 methylation /lymphatic emboli status			0.001
	High/absent (*n* = 58)	Reference	
	Low/present (*n* = 28)	3.128 (1.523–6.424)	0.002
	Low/absent (*n* = 44)	1.113 (0.460–2.695)	0.812
	High/present (*n* = 42)	0.761 (0.237–2.443)	0.647
Gross type			0.073
	Mass forming (*n* = 141)	Reference	
	Periductal infiltrative (*n* = 8)	0	0.972
	Intraductal growth (*n* = 18)	0.141 (0.019–1.032)	0.054
	Mixed (*n* = 5)	3.013 (0.885–10.254)	0.078
T staging			0.152
	pT1 (*n* = 48)	Reference	
	pT2 (*n* = 52)	0.579 (0. 220–1.526)	0.270
	pT3 (*n* = 47)	0.371 (0.124–1.114)	0.077
	pT4 (*n* = 25)	0.233 (0.056–0.971)	0.046
N staging	pN0 (*n* = 133)		
	pN1 (*n* = 39)	2.456 (1.341–4.497)	0.004
Perineural invasion	Absent (*n* = 118)		
	Present (*n* = 54)	0.321 (0.147–0.699)	0.004
Differentiation			0.577
	Well (*n* = 23)	Reference	
	Moderate (*n* = 94)	3.495 (0.332–36.809)	0.298
	Poor (*n* = 55)	3.493 (0.328–37.217)	0.300

## Conclusions

In summary, we assessed LINE-1 methylation levels in a total of 172 cases of ICC using PCR pyrosequencing and elucidated whether LINE-1 methylation status was correlated with clinicopathological features of ICCs. We found that tumoral LINE-1 hypomethylation was an independent prognostic factor of outcomes in ICC patients, heralding decreased survival. Further study is required to validate our findings of tumoral LINE-1 hypomethylation as a prognostic marker.

## Additional files

Additional file 1: A. Scatter plot of LINE-1 methylation and percentage of non-neoplastic stroma cells in dissected tumor areas. B. Box plot of LINE-1 methylation by grouping ICCs into 3 subsets according to percentage of non-neoplastic stroma cells. Analysis of the relationship between LINE-1 methylation level and stroma ratio of tumor area did not show significant correlation between them. No significant difference was seen in tumoral LINE-1 methylation level between four subsets of ICCs according to percentage of non-neoplastic cells (<10%, 10–19%, 20–30%, >30%). (JPEG 307 kb)

Additional file 2: Cancer-specific survival rates with performance of tumoral LINE-1 hypomethylation in ICCs with stroma cell ratio of ≤15% (A) and in ICCs with stroma cell ratio of >15% (B). ICC cases were grouped into two subsets according to their stroma cell ratio (≤15% (*n* = 90), and, >15% (*n* = 82)) and then evaluated regarding prognostic potential of low methylation status of LINE-1 in each subset. Prognostic significance of low methylation status of LINE-1 was seen in two subsets. (JPEG 232 kb)

## Abbreviations

ECC: Extrahepatic cholangiocarcinoma
ICC: Intrahepatic cholangiocarcinoma
IG: Intraductal growth
LINE-1: Long interspersed element-1
MF: Mass-forming
PI: Periductal infiltrative
TNM: Tumor, node, and metastasis
