# Supplementary figures and images for: Tumoral LINE-1 hypomethylation is associated with poor survival of patients with intrahepatic cholangiocarcinoma
Source: BMC Cancer. 2017 Aug 29;17:588. doi: 10.1186/s12885-017-3595-8 (PMC5576385; doi:10.1186/s12885-017-3595-8)

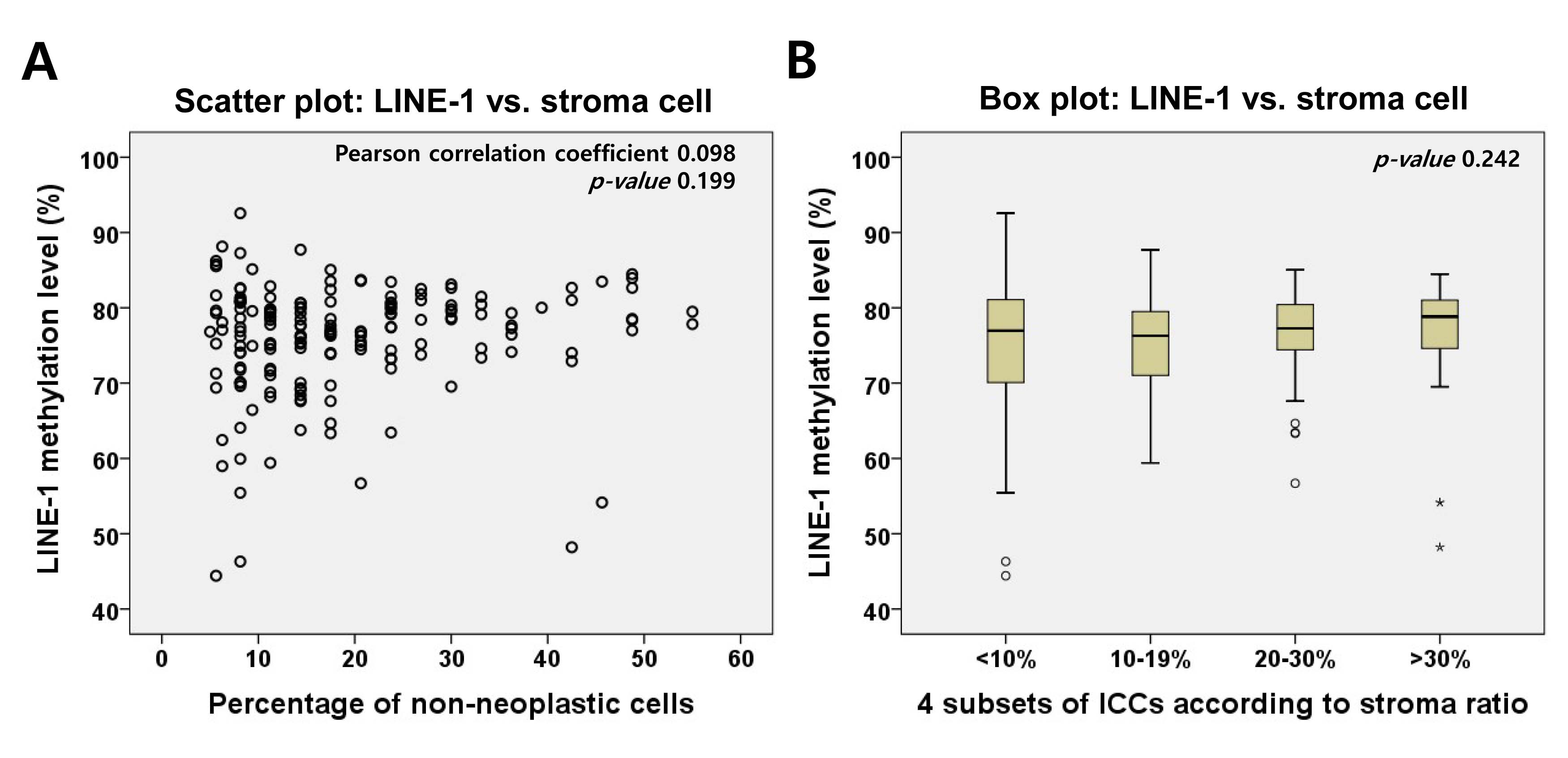

Supplement: Supplementary file 1 — A. Scatter plot of LINE-1 methylation and percentage of non-neoplastic stroma cells in dissected tumor areas. B. Box plot of LINE-1 methylation by grouping ICCs into 3 subsets according to percentage of non-neoplastic stroma cells. Analysis of the relationship between LINE-1 methylation level and stroma ratio of tumor area did not show significant correlation between them. No significant difference was seen in tumoral LINE-1 methylation level between four subsets of ICCs according to percentage of non-neoplastic cells (<10%, 10–19%, 20–30%, >30%). (JPEG 307 kb) [file 12885_2017_3595_MOESM1_ESM.jpg]

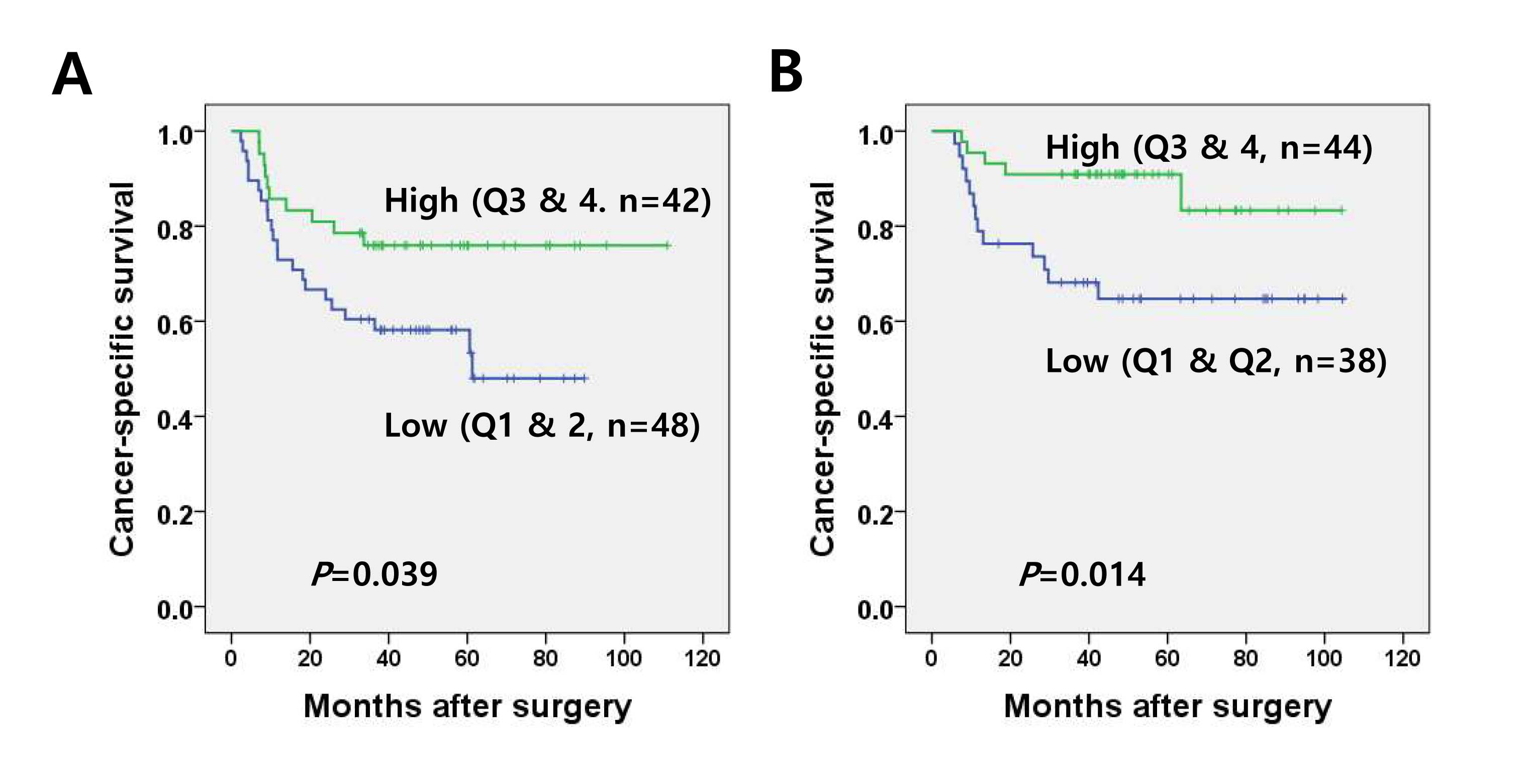

Supplement: Supplementary file 2 — Cancer-specific survival rates with performance of tumoral LINE-1 hypomethylation in ICCs with stroma cell ratio of ≤15% (A) and in ICCs with stroma cell ratio of >15% (B). ICC cases were grouped into two subsets according to their stroma cell ratio (≤15% (n = 90), and, >15% (n = 82)) and then evaluated regarding prognostic potential of low methylation status of LINE-1 in each subset. Prognostic significance of low methylation status of LINE-1 was seen in two subsets. (JPEG 232 kb) [file 12885_2017_3595_MOESM2_ESM.jpg]
